# Combined electrohydraulic and holmium: YAG laser ureteroscopic nephrolithotripsy of large (>2 cm) renal calculi

**DOI:** 10.4103/0970-1591.44261

**Published:** 2008

**Authors:** Albert J. Mariani

**Affiliations:** Department of Urology, University of Hawaii John A. Burns School of Medicine, Kaiser Medical Center, 3288 Moanalua Road, Honolulu, HI 96819, USA

**Keywords:** Electrohydraulic, holmium:YAG laser, kidney calculi, lithotripsy, ureteroscopy

## Abstract

Percutaneous nephrolithotripsy (PCL) is a standard treatment for renal calculi >2 cm. Modern flexible ureteroscopes and accessories employing the complementary effects of electrohydraulic lithotripsy (EHL) and Ho:YAG laser lithotrites can treat these renal calculi in a minimally invasive fashion with similar or superior results.

**Objective:**

To assess the safety and efficacy of ureteroscopic nephrolithotripsy monotherapy for the management of >2 cm renal calculi in the community setting.

**Materials and Methods:**

Fifty nine patients with 63 renal calculi ranging from 20 to 97 mm (mean 44 mm) in length and 175 to 3300 mm2 (mean 728 mm2) area underwent staged ureteroscopic nephrolithotripsy monotherapy. Obesity (BMI > 30) was present in 54% and 19% were morbidly obese (BMI > 40). An infectious etiology was present in 49% and hard stone components in 89%. All patients presented with hematuria, pain, and/or recurrent urinary tract infection (UTI). Lithotripsy was performed with a single deflection flexible ureteroscope and predominantly EHL. Laser drilling was employed (n = 6) to weaken very hard stones prior to EHL. Low intrarenal pressure was maintained by continuous bladder drainage and placement of a stiff safety wire. Visibility was maintained using manual pulsatile irrigation.

**Results:**

All patients were rendered pain and infection-free. No patient required a blood transfusion and there was no change in serum creatinine. Mobile stone-free status was achieved in 60/63 (95%) with a mean of 1.7 nephrolithotripsy stages and 0.38 secondary or ancillary procedures. Outpatient management was sufficient for 121/131 (92%) of the procedures. Operative time averaged 46 min/stage and 79 min/calculus. Complications included endotoxic shock (3), fever (5), ureteral fragments requiring treatment (11), delayed extubation (2), delayed pneumonia (1), and urinary retention (1).

**Conclusion:**

Staged ureteroscopic nephrolithotripsy of large renal calculi is feasible with low morbidity and stone clearance rates that compare favorably with PCL. It has largely replaced PCL at this institution.

## INTRODUCTION

Untreated, usually infected, staghorn calculi are likely to eventually cause renal destruction and/or life-threatening sepsis.[1,2] The 2005 Americal Urological Association (AUA) guideline for the management of staghorn calculi recommended percutaneous nephrolithotripsy (PCL) as the first-line treatment for calculi >500 mm^2^. PCL was noted to have a clearance rate of approximately 78% (range 74-83%) requiring 1.9 procedures with possible under-reporting. There was an 18% (range 14-24%) transfusion rate, and a 15% (range 7-27%) significant complication rate including acute renal loss, chronic renal failure, vascular injury, colonic injury, hydrothorax, pneumothorax, prolonged urine leakage, steinstrasse, impacted ureteral calculi, ureteral colic requiring hospitalization, pyelonephritis, sepsis, deep venous thrombosis, and pulmonary embolus.[3] It was felt that there was insufficient data to recommend ureteroscopy except as an adjunctive procedure to remove fragments. Concerns were raised about poor stone clearance rates, multiple procedures, and long operative times.

Nevertheless ureteroscopic nephrolithotripsy using both electrohydraulic lithotripsy (EHL)[4] and Ho:YAG laser lithotrites[5] or both[6] has been demonstrated to be safe and effective. The current study was performed to test the hypothesis that modern flexible ureteroscopes employing the complementary effects of EHL and Ho:YAG laser lithotripsy can treat staghorn calculi in a minimally invasive fashion with similar or superior results to PCL in a community hospital setting.

## MATERIALS AND METHODS

### Technique

The study received institutional review board approval. Bacteruric patients were treated for three days prior to the procedure with oral antibiotics guided by sensitivities. Prior to the procedure they were administered intravenous antibiotics - usually ampicillin and gentamicin. Under general anesthesia patients were positioned in a modified lithotomy position with the ipsilateral leg straightened to reduce the psoas bulge and in Trendelenberg to allow stone fragments to fall into more accessible upper pole calyces.

Under fluoroscopic guidance a 0.038″ hydrophilic guidewire was passed into the renal pelvis. A 6/10 French dual lumen ureteral stent was passed over the guidewire into the renal pelvis to dilate the ureter and to pass a second stiff 0.038″ hydrophilic guidewire which was secured to the drapes as a safety wire and ureteral straightening device. This straightening plus the tenting effect on the ureter improved ureteral drainage. The stiffness of the secured stiff hydrophilic guidewire prevented distal migration.

A current generation, single active deflection, flexible ureteroscope (DUR-8) (ACMI Corporation, Southborough, MA, USA) was passed over a standard hydrophilic guidewire under fluoroscopic guidance into the renal pelvis. A 12-Fr Foley catheter was placed to straight drainage to maintain low bladder and collecting system pressures.

The ureteroscope guidewire was replaced with a 1.9-Fr EHL probe which was positioned 5 mm from the ureteroscope tip to protect the ureteroscope and 1-3 mm from irregularities on the stone to maximize the kinetic energy transfer to the calculus.[7] The tip of the EHL probe was continuously visualized to avoid intra pelvic or papillary injury. The best results were achieved using an AEH-3 EHL generator (ACMI Corporation, Southborough, MA, USA) at <0.5 s discharges usually at the 95 (hard setting). After initial fragmentation, continued fragmentation was often achieved with lower generator settings. The lowest setting that would efficiently fragment the calculus was used to minimize the risk of soft tissue injury and to prolong the useful life of the probe. Manual piston irrigation (Irri-flow II) (ACMI Corporation, Southborough, MA, USA) was employed to improve visualization, sweep away clots, and fragments, and estimate fragment size on the basis of fragment mobility. Video was employed throughout the procedure to facilitate staff assistance.

For very hard stones (six cases) resistant to EHL, a 200-mm Ho:YAG laser fiber was employed to drill holes in the calculus. This sufficiently weakened the calculus such that discharge of the EHL probe into the laser defect (“fire in the hole”) resulted in good initial fragmentation.

When stone fragments appeared to be <3 mm on the basis of mobility with irrigation, fluoroscopic appearance, and using the guidewire (1 mm) and the EHL probe (0.7 mm) as visual size gauges, the procedure was terminated. The ureter was inspected during withdrawal of the ureteroscope. A 7-Fr double pigtail stent was placed. Prior to removing the cystoscope after draining the bladder 15 mg of ketorolac in 30 cc of saline was injected intravesically to reduce postoperative discomfort.[8] Patients were monitored closely for sepsis a minimum 6 h in postanesthesia recovery after the procedure, then discharged if they remained asymptomatic. Patients were discharged with a 14-day supply of 0.4 mg tamsulosin to be taken prior to bedtime to promote minimally symptomatic fragment passage.[9]

Two to seven days postoperatively the stent was removed in the clinic using the tether suture. Patient with infection stones were treated with oral antibiotics for five to seven days, then placed on low-dose suppressive antibiotics until stone clearance. Patients were prescribed potassium citrate 20 mEq/day three to four times per day orally for six weeks[10] and advised to force fluids and sleep on their side with the operative side up. Stone debris was collected intraoperatively or postoperatively and sent for analysis. Most stone debris passed within two weeks of each stage. Imaging was performed at 10-14 days and four to eight weeks. If steinstrasse was present the next stage of the procedure was scheduled within two weeks - if not then within six weeks. The endpoint was achieved at six weeks after the last stage when diagnostic imaging studies or ureteroscopy confirmed the presence or absence of residual calculi. A metabolic stone evaluation was performed on patients after stone clearance. Patients were treated long-term with specific medical therapy.

### Steinstrasse management

In two of three cases, both the flexible and stiff (safety) hydrophilic guidewires traversed the steinstrasse without difficulty. In one case the flexible hydrophilic guidewire, which was placed first, would not traverse the steinstrasse. With the patient in Trendelenberg position, the wire was advanced into the steinstrasse until buckling occurred. A 6/10-Fr dual lumen ureteral catheter was then placed over the wire up to the steinstrasse. Through the remaining port, 5 ml of saline was injected. This realigned the steinstrasse and allowed easy passage of both the guidewires. The ureteroscope was positioned to the level of the steinstrasse in the standard fashion. If a lead fragment was present and impacted, it was fragmented using the Ho:YAG laser. If loose steinstrasse was present it was pushed into a dilated portion of the ureter where EHL could be safely employed. The combination of EHL fragment disruption and irrigation allowed advancement of the ureteroscope into the renal pelvis where the procedure continued as the next stage of the nephrolithotripsy.

### Prevention of steinstrasse

Early in the series it was noted that steinstrasse tended to occur at the same levels in the ureter in the same patient suggesting an occult ureteral stricture. Since adding balloon dilation of the ureter for all patients with calculi >3 cm prior to the first lithotripsy the incidence of steinstrasse appears to have been much reduced. Numbers remain too small for statistical analysis.

## RESULTS

Between January 2001 and October 2006, 59 patients with 63 renal calculi >2 cm in length were treated with ureteroscopic nephrolithotripsy after obtaining informed consent. Three additional patients were not treated with ureteroscopic nephrolithotripsy. Two patients met the absolute exclusion criteria of an uncontrolled urinary tract infection (UTI) and an inaccessible ureter. One patient met the relative exclusion criterion of poor renal function defined as a creatinine of >4.0 mg/dl (nl.: 0.5-1.2 mg/dl). These patients were treated by other modalities. Three month follow-up was available on all patients.

All patients presented with pain and/or, hematuria and/or recurrent UTI. Obesity (BMI > 30) was present in 32/59 (54%) of the patients while 11/59 (19%) were morbidly obese (BMI > 40). The mean calculus length was 44 mm (range 20-97 mm). The mean area on kidneys ureters bladder (KUB) plain film was 728 mm^2^ (range 560-2425 mm^2^). There were 27 three calyx staghorn calculi. Infection was present in 49% of the patients and 89% of the calculi contained hard components of apatite and/or calcium oxalate monohydrate. There was an average of 1.7 nephrolithotripsy stages per calculus and an average operative time of 46 min per stage for an average total operating time of 79 min per calculus. Of the 131 procedures, 121 (92%) were outpatient. Surgical success, defined as clearance of all mobile stone fragments, was achieved in 60/63 (95%). Follow-up diagnostic ureteroscopy (n = 27), KUB (n = 16), excretory urogram(ExU) (n=9) computerized tomogram (n=8), or renal ultrasound (n=3) were used to determine the stone-free status. In one case an 11 × 14 mm^2^ fragment was retained in an anterior calyx inaccessible to the ureteroscope and impervious to electrohydraulic shock wave lithotripsy (SWL). In another case there were some 2 mm fragments present in a lower pole calyx on a follow-up RUS. Unfortunately, shortly thereafter the patient developed a rapidly progressive gastric carcinoma which made further treatment inappropriate. In one anticoagulated patient, there was a 7 mm accumulation of 1-3 mm lower pole fragments that required basket removal one year postoperatively for the management of hematuria.

Three patients developed endotoxic shock and required intensive care unit (ICU) admission and supportive therapy. All cultures were negative. Five patients developed postoperative fever and chills. All were admitted for overnight observation. Cultures were negative in four. One patient who had multiple previous antibiotic courses had developed a postoperative fungemia which responded to parenteral antifungal therapy. The preoperative urine culture and sensitivities (UC&S) cultures were negative. One 90-year-old patient developed pneumonia requiring hospitalization three days after discharge. Eleven patients developed either asymptomatic or minimally symptomatic steinstrasse. Four required ancillary ureteroscopic procedures to clear the steinstrasse while in the remaining patients the steinstrasse cleared spontaneously or was removed at the next scheduled nephrolithotripsy stage. There were two cases of delayed extubation associated with sleep apnea. There was one case of urinary retention which was managed with Foley catheterization. The patient had a successful void trial three days postoperatively. Collectively these complications resulted in 46 days of hospitalization (0.78 days/patient), nine unplanned clinic and three emergency department visits.

No patient required a blood transfusion. A pre- and post-treatment serum creatinine was available for 44/59 patients. Thirteen patients had a creatinine increase of 0.1-0.3 mg/dl while 28 had a decrease of 0.1-0.9 mg/dl for a mean difference of −0.08 mg/dl which approximates the precision of the test. All patients were rendered pain, hematuria, and infection-free.

## DISCUSSION

In 1990 Aso *et al.* published a landmark series of 34 staghorn calculi treated with flexible ureteroscopic EHL monotherapy. There was a 50% stone clearance rate, a 50% incidence of fever >38 °C and 12% incidence of sepsis.[8] Since then there has been remarkable progress in ureteroscopy. Modern flexible ureteroscopes are smaller and more durable, have greater flexibility, improved optics, and larger more durable working ports. This has been paralleled by the development of the Ho:YAG laser, improved guidewires, baskets, stents, and other ureteroscopic accessories. Finally a better understanding of ureteral and urolithiasis physiology has resulted in improved medical therapy for stone pain control (ketoralac),[9] stone passage (tamsulosin),[10] and recurrence (potassium citrate).[11]

Two miniature, flexible lithotrites with complementary properties enable successful ureteroscopic lithotripsy. The Ho:YAG laser provides precise thermal drilling powerful enough to vaporize the hardest of calculi. Significant tissue injury and bleeding is minimized by the near contact nature of the laser and the small depth of penetration (0.5 mm). Generated stone fragments are finer.[12]

The EHL lithotriptor generates a 3-6 kV difference between two electrodes. This causes a spark that results in a plasma channel and explosive vaporization of the water medium. This in turn generates a shock wave followed by a cavitation bubble. The collapse of the cavitation bubble causes a secondary shock wave leading to the formation of high-speed microjets. The shock wave and the high-speed microjets disrupt the crystalline structure of a calculus. Maximal kinetic energy transfer is achieved at a 1-3 mm probe tip distance from the surface of the calculus.[13] Because of the longer effective range (1-3 mm vs. 0.5 mm) the EHL fragments much larger volumes of stone per unit of time than the Ho:YAG laser thus shortening operative time. Another advantage is flexibility. The 1.9-Fr EHL probe permits greater ureteroscope deflection improving access to all parts of the collecting system. Unlike a Ho:YAG laser fiber, a 1.9-Fr EHL probe can be gently passed down a prepositioned, flexed ureteroscope with minimal risk to the working channel further improving access. While the Ho:YAG laser easily cuts through a guidewire or basket, this is unlikely with EHL. Eye protection is not required with EHL. Finally, because of the simplicity of the technology, the cost and maintenance of EHL is much less than for the Ho:YAG laser.

For calculi impervious to the EHL the Ho:YAG laser can create holes thereby weakening the structure so that the chief EHL advantage - high bulk fragmentation per unit time - can be achieved. In this series the bulk of stone material was disintegrated using EHL. Only six required “holing” with the Ho:YAG laser. In all six cases, discharging the EHL in the holes initiated a fragmentation that could be continued to completion. Because of the 1-3 mm shock wave of EHL care must be taken not to discharge against soft tissues, the tip should be visualized at all times. Because there is typically more bleeding when using the EHL, attention must be given to providing good vision. This was achieved by employing manual pulsatile irrigation, Foley catheter bladder drainage, and a stiff hydrophilic ureteral safety wire.

Like SWL ureteroscopic nephrolithotripsy has the goal of reducing a calculus too large to pass spontaneously to fragments which will pass. Unlike SWL, ureteroscopic nephrolithotripsy permits visual control over the size of generated stone fragments. It is not necessary to reduce the stone to dust which would increase procedure time and the amount of energy discharged into the renal pelvis. Nor is it necessary to remove the multitude of stone fragments generated. This would prolong the procedure and increase the risk of basket complications. It is well established that calculi < 5 mm will pass spontaneously - especially after stenting.[14] Fragment passage after SWL is usually minimally symptomatic. That was the experience in this series.

Success was defined as the clearance of mobile calcifications. Thirty-three patients had calcifications overlying the kidney on KUB. Ureteroscopy confirmed that these were fixed calcifications. They often extended deep into the renal parenchyma suggesting that diagnostic ureteroscopy may in fact be the gold standard of determining success after the surgical treatment of renal calculi. As most of these plaques were papillary, surgically excavating with either lithotrite did not seem prudent. Aggressive medical therapy would seem to be more appropriate. Indeed the rather extensive parenchymal calcifications noted in Case eight had resolved at the one year follow-up with aggressive medical therapy. Removing asymptomatic, noncommunicating calyceal diverticular calculi would also be overly aggressive.

Retained ureteral fragments (steinstrasse) occur after both SWL and PCN. In this series 5/8 (63%) with calculi >75 mm required steinstrasse management. This is not surprising because as the linear length of a calculus increases the volume increases exponentially. In all five cases the steinstrasse was minimally symptomatic with no unplanned or emergency department visits. As Resim *et al.*[10] notedthe discomfort of steinstrasse after SWL can be alleviated by prophylactic treatment with tamsulosin. Tamsulosin also facilitates passage of distal ureteral calculi.[15,16]

Patients should be infection-free prior to the procedure. Unfortunately preoperative bladder urine cultures are a poor predictor of infection in the stone or in the upper tract urine.[16] For this reason patients with a history of recurrent UTIs should be pretreated with oral antibiotics prior to a procedure. Patients should be monitored closely after ureteroscopic nephrolithotripsy. O'Keefe noted a 1.3% incidence of septic shock after approximately 700 percutaneous or endoscopic procedures with a 66% mortality. In that study septic shock presented within 6 h and in most cases within 2-3 h.[17] Preoperative and intraoperative antibiotic therapy cannot be expected to control endoxemia which can result from the release of endotoxins contained within the stone structure during the lithotripsy.[18] Supportive treatment early in the course of septic shock is the only effective treatment of this potentially lethal complication. While 90% of the procedures in this series were performed as outpatient, in all cases arrangements should be made for the prompt admission of any patient experiencing rigors and/or fever or significant changes in closely monitored vital signs.

Every attempt should be made to clear all mobile fragments before declaring surgical success.[19] Potassium citrate has been demonstrated to improve the fragment clearance rate after SWL.[10] Finally, medical evaluation and management have been demonstrated to control active stone formation after SWL.[20]
